# Comparative Effect of Cardamom (*Elettaria cardamomum*) and Clove (*Syzygium aromaticum*) on Body Weight and Nutritional Status in Obese Experimental Animals

**DOI:** 10.1155/jnme/8894025

**Published:** 2025-11-25

**Authors:** El-Sayed H. Bakr, Raneem Waleed Mahboob, Dalia Saleh Al-Zahrani, Lojain Osama Sedyou, Manar Osama Msri, Areej A. Almuraee, Ziad T. Kishmira, Mohammad A. Althubiti, Khalid Waleed Mahboob

**Affiliations:** ^1^Department of Nutrition and Food Sciences, Menofia University, Shebein El-Kom, Egypt; ^2^Department of Quality and Development, Batterjee Medical College, Dammam 32313, Saudi Arabia; ^3^Department of Clinical Nutrition, Faculty of Applied Medical Sciences, Umm Al-Qura University, Holy Makkah, Saudi Arabia; ^4^Department of Laboratory Analysis, KAFAAT Medical Center, Holy Makkah, Saudi Arabia; ^5^Biochemistry Department, Faculty of Medicine, Umm Al-Qura University, Holy Makkah, Saudi Arabia; ^6^Department of Basic Science, Faculty of Basic Science and Health Professions College, King Saud bin Abdulaziz University for Health Sciences, Jeddah, Saudi Arabia

**Keywords:** cardamom, clove, nutritional status, obesity, rats

## Abstract

**Background:**

Obesity is a serious health concern associated with many diseases. Studies have found that medicinal herbs have potential leads to treatment. Therefore, clove and cardamom extracts are known to have a positive effect on obesity management and reducing obesity-related risks.

**Aims:**

This study aimed to compare the effect of cardamom (*Elettaria cardamomum*) and clove (*Syzygium aromaticum*) on the body weight and nutritional status of obese experimental animals.

**Methods:**

Twenty male Sprague–Dawley albino rats were divided into four groups (five rats each) as follows; The first was the negative control group, the second was the positive control group (obese rats), the third group was obese with oral administration of 1 mg/kg b.wt. of cardamom alcoholic extract, and the fourth group was obese with oral administration of 1 mg/kg b.wt. of clove alcoholic extract. At the end of the experiment (28 days), rats were mercy sacrificed; the body weight gain, food intake, and feed efficiency ratio were measured; and TC, TG, HDL, LDL and VLDL were analyzed.

**Results:**

Rats treated with cardamom and clove extracts showed a significant decrease in body weight gain compared to the positive control group, with a higher significant reduction in cardamom, in addition, for enhancing nutritional status.

**Conclusion:**

Cardamom and clove improve the body weight and nutritional status, as well as improve food intake and feed efficiency ratio, showing a higher effect of the cardamom extract.

## 1. Introduction

Obesity is defined as an excessive accumulation of fat that threatens human health. A body mass index (BMI) over 25 is classified as overweight, and a BMI over 30 is classified as obesity. This problem has become epidemic because over 4 million individuals die annually as a result of being overweight or obese. The prevalence of obesity is still increasing, rising from 4% to 18% worldwide among children and adolescents aged 5–19; this problem tends to be more common in urban regions of low- and middle-income countries [1]. People who suffer from being overweight and obesity face an increased risk of chronic diseases, as well as psychological and social problems. To prevent and treat overweight and obesity, there are many interventions, mainly nutritional interventions. Many medicinal herbs and their derivatives have been reported to prevent obesity and its associated diseases. The effectiveness of cardamom and clove herbal extracts has been found in developing antiobesity agents to reduce body weight [2]. *Elettaria cardamomum* (commonly known as cardamom), Zingiberaceae family, is a perennial herbaceous plant that grows to a height of 400–500 cm. Cardamom is a popular spice in the Indian subcontinent, where it has long been used in cooking and traditional medicinal practices. Cardamom has the therapeutic effects of its pharmacological properties, including being rich in antioxidant, anti-inflammatory, antibacterial, antimutagenic, antidiabetic, cardioprotective, hepatoprotective, and chemoprotective properties [3]. *Syzygium aromaticum* plant (commonly known as clove), Myrtaceae family, is a dried flower bud that belongs to the Myrtaceae family [4]. The clove tree consists of leaves and buds and begins producing flowering buds 4 years after planting. It is considered one of the most important traditional spices used in foods. It has been used to preserve food and has medicinal properties. It is rich in many phytochemicals and has antioxidant, antimicrobial, and antiobesity properties. It has great potential in many fields, including agriculture, pharmacy, and food [5]. Accordingly, this investigation aimed to compare the effect of cardamom and clove on the body weight and nutritional status in obese experimental animals.

## 2. Materials, Rats, and Methods

### 2.1. Materials and Rats

#### 2.1.1. Investigated Samples

Cardamom and clove were obtained from the local market, Makkah, Saudi Arabia.

#### 2.1.2. Rats

Twenty Sprague–Dawley male albino rats with an average weight of 200 ± 10 g were obtained from the Department of Medical Biochemistry, Faculty of Medicine, Umm Al-Qura University, Holy Makkah, Saudi Arabia.

#### 2.1.3. Basal Diet

The basic diet consists of casein (14%), soybean oil (4%), sucrose (10%), fiber (5%), mineral mixture (3.5%), vitamin mixture (1%), choline (0.25%), l-cystine, and cornstarch (62.07%) [6].

#### 2.1.4. High-Fat Diet (HFD)

The HFD consists of 4.8 g/day fat, 3.2 g/day protein, 6.4 g/day carbohydrates, 0.64 g/day fiber, 0.16 g/day vitamin mixture, and 0.64 g/day mineral mixture [7].

### 2.2. Methods

#### 2.2.1. Preparation of Extracts

Alcoholic cardamom extract was prepared by grinding fresh cardamom and using 50-g powder in an alcohol distiller rotary evaporator at a temperature of 70°C for 30 min, and was given to the rats orally. Meanwhile, alcoholic clove extract was prepared in the same way as cardamom extract, which was prepared by grinding cloves and using 50 g of clove powder in a rotary alcohol distiller at a temperature of 70°C for 30 min, and was given to the rats orally.

#### 2.2.2. Experimental Design

Rats were initially fed a basal diet for a week, after which the rats were divided into four groups (5 rats each) as follows: The first group was the negative control group (−ve) which fed on a basal diet. The second group was the positive control group (+ve) which fed on a HFD. The third group was the first experimental group which fed on a HFD and then orally administered with 1 mg/kg b.wt. of alcoholic cardamom extract. The fourth group was the second experimental group which fed on a HFD and then orally administered with 1 mg/kg b.wt. of alcoholic clove extract.

At the end of the experiment (28 days), the rats were sacrificed using diethyl ether anesthesia, and the required blood and organs were collected and then disposed of in the designated container.

#### 2.2.3. Biological and Biochemical Analyses

During the experimental period, body weight gain (BWG) and food intake (FI) were measured, and the feed efficiency ratio (FER) was calculated. At the end of the experiment, blood samples were collected from the hepatic portal vein to determine the levels of triglyceride (TGL) and total cholesterol (TC), high-density lipoprotein (HDL), low-density lipoprotein (LDL), and very LDL (VLDL). Moreover, organs (liver, kidneys, spleen, lungs, and heart) were obtained to determine their weight.

#### 2.2.4. Statistical Analysis

Statistical analysis was performed using the SPSS program (Ver. 22). Results were expressed as mean ± SD from five rats in each group, which was performed using a one-way analysis of variance (ANOVA) followed by Dunnett's test for multiple comparisons, with the significance level chosen at *p* < 0.05.

#### 2.2.5. Ethics Statement

Ethical approval was obtained by the Scientific Research Ethics Committee, Committees of the College Vice-Deanship for Postgraduate Studies, Faculty of Medicine, Umm Al-Qura University, Mecca, Saudi Arabia (Approval no. HAPO-02-K-012-2023-12-1927).

## 3. Results and Discussion

### 3.1. Results

#### 3.1.1. Chemical Composition of Cardamom and Clove

Cardamom and cloves contain many active ingredients that contribute to their action and properties. The major components of cardamom were found to be α-terpinyl acetate, 1,8-cineole linalool, and other monoterpenes, such as camphene and carvacrol, which are present in less than 0.1% levels [8]. Meanwhile, the main components of clove are eugenol and oleanolic acid, and other compounds include eugenyl acetate, beta-caryophyllene, alpha-humulene, sterols, flavonoids, methyl amyl ketone, and other volatile chemicals [9, 10].

#### 3.1.2. Comparative Effect of Cardamom and Clove on BWG in Obese Rats

Data listed in Table 1 show the effect of cardamom and clove on BWG in obese rats.

It could be observed for obese rats without treatment (C +ve group) that BWG was 53.00 ± 0.47 compared to 30.00 ± 0.79 g/28 days in the negative-control normal rats (*p* < 0.05). These results denote a significant increase in BWG of obese rats compared to normal rats. All rats orally administered with the alcohol extract of cardamom and clove showed a significant decrease in BWG as compared to the positive control group, which were 41.13 ± 0.35 and 48.18 ± 0.73 g/28 days, respectively. Moreover, the cardamom group showed the highest significant decrease in body weight compared to the clove group.

#### 3.1.3. Comparative Effect of Cardamom and Clove on FI and the FER in Obese Rats

Data listed in Table 2 show the effect of cardamom and clove on FI and FER in obese rats.

It could be observed for obese rats without treatment (C +ve group) that FI was 16.13 ± 0.88 compared to 11.37 ± 0.08 g/28 days in negative-control normal rats (*p* < 0.05).

These results indicate a significant increase in FI in obese rats compared to normal rats. All rats orally administered with the alcohol extract of cardamom and clove showed a significant decrease in FI as compared to the positive control group, which were 13.57 ± 0.27 and 15.18 ± 0.57 g/28 days, respectively. Moreover, the cardamom group showed the highest significant decrease in FI when compared to the clove group.

As for the group of obese rats without treatment (Group C +ve), the FER was 0.117 ± 0.002 compared to 0.094 ± 0.001 for 28 days in normal rats (*p* < 0.05). These results indicate a significant increase in the FER in obese rats compared to normal rats. All rats that were administered orally with the alcoholic extract of cardamom and clove showed a significant decrease in FER compared to the positive control group, which amounted to 0.108 ± 0.002 and 0.113 ± 0.001 for 28 days, respectively. Moreover, the cardamom group showed the highest significant decrease in FER compared to the clove group.

#### 3.1.4. Comparative Effect of Cardamom and Clove on the Relative Liver and Kidney Weights to Body Weight Ratio in Obese Rats

Data recorded in Table 3 show the effect of cardamom and clove on the relative liver and kidney weights to body weight ratio in obese rats.

It could be observed for obese rats without treatment (C +ve group) that their relative liver and kidney weights to body weight ratio were 3.04 ± 0.03 and 0.87 ± 0.04%, compared to 2.57 ± 0.02 and 0.68 ± 0.01%, respectively, in negative-control normal rats (C −ve group). According to the results, obese rats had a significantly higher relative liver and kidney weight to body weight ratio in obese rats compared to normal rats. All rats orally administered with alcohol extracts of cardamom and clove showed a significant decrease in relative liver weight to body weight ratio, compared to the positive control group, which were 2.61 ± 0.01 and 2.87 ± 0.03%, respectively, and a significant decrease in relative kidney weight to body weight ratio, which were 0.71 ± 0.02 and 0.78 ± 0.03%, respectively. Moreover, the cardamom group showed the highest significant decrease in the relative liver and kidney weight to body weight ratio when compared to the clove group.

#### 3.1.5. Comparative Effect of Cardamom and Clove on Relative Heart, Lungs, and Spleen Weight to Body Weight Ratio in Obese Rats

Data listed in Table 4 show the effect of cardamom and clove on the relative heart, lungs, and spleen weight to body weight ratio in obese rats.

It could be observed for obese rats without treatment (C +ve group) that their relative heart, lungs, and spleen weight to body weight ratio were 0.43 ± 0.05, 0.71 ± 0.04, and 0.61 ± 0.02%, respectively, compared to 0.33 ± 0.02, 0.52 ± 0.01, and 0.23 + 0.03%, in negative-control normal rats (C −ve group). According to these results, obese rats had a significantly higher relative heart, lungs, and spleen weight to body weight ratio when compared to normal rats. When compared to the positive control group, all rats that received oral administration of the alcoholic cardamom and clove extract showed a significant decrease in relative heart weight to body weight, which were 0.37 ± 0.04 and 0.39 ± 0.03%, respectively, and a significant decrease in relative lung weight to body weight, which were 0.57 ± 0.03 and 0.61 ± 0.02%, and also showed a significant decrease in relative spleen weight to body weight ratio, which were 0.31 ± 0.01 and 0.41 ± 0.04%, respectively. Meanwhile, the cardamom group showed the highest significant decrease in the relative heart, lungs, and spleen weight to body weight ratio compared to the clove group.

#### 3.1.6. Comparative Effect of Cardamom and Clove on TGL and TC in Obese Rats

Data recorded in Table 5 show the effect of cardamom and clove on TGL and TC in obese rats.

It could be observed for obese rats without treatment (C +ve group) that TGL and TC were 1.99 ± 0.06 and 2.08 ± 0.05 mmo/L, respectively, compared to 0.76 ± 0.02 and 1.15 ± 0.02 mmol/L, in negative-control normal rats (C −ve group). According to these results, obese rats had significantly higher TGL and TC compared to normal rats. When compared to the positive control group, all rats that received oral administration of the alcoholic cardamom and clove extract showed a significant decrease in TGL, which were 0.98 ± 0.01 and 1.17 ± 0.03 mmol/L, respectively, and a significant decrease in TC, which were 1.13 ± 0.03 and 1.144 ± 0.04 mmol/L, respectively. Moreover, the cardamom group showed the highest significant decrease in TGL and TC compared to the clove group.

#### 3.1.7. Comparative Effect of Cardamom and Clove on HDL-Cholesterol (HDL-c), LDL-cholesterol (LDL-c), and VLDL-Cholesterol (VLDL-c) in Obese Rats

Data presented in Table 6 show the effect of cardamom and clove on HDL-c, LDL-c, and VLDL-c in obese rats.

It could be observed for obese rats without treatment (C +ve group) that the mean values of HDL-c, LDL-c, and VLDL-c were 0.43 ± 0.06, 1.25 ± 0.023, and 0.398 ± 0.03 mmol/L, respectively, compared to 0.89 ± 0.02, 0.11 ± 0.013, and 0.152 ± 0.05 mmol/L, in negative-control normal rats (C −ve group). According to these results, obese rats had significantly higher LDL-c and VLDL-c when compared to normal rats. When compared to the positive control group, all rats that received oral administration of the alcoholic cardamom and clove extract showed a significant increase in HDL-c, which were 0.79 ± 0.01 and 0.72 ± 0.03 mmol/L, respectively, and a significant decrease in LDL-c, which were 0.14 ± 0.017 and 0.19 ± 0.026 mmol/L, and also showed a significant decrease in VLDL-c, which were 0.196 ± 0.03 and 0.234 ± 0.07 mmol/L, respectively. Moreover, the cardamom group showed the highest significant increase in HDL-c and a decrease in LDL-c and VLDL-c compared to the clove group.

### 3.2. Discussion

Global epidemiological evidence indicates that the incidence of obesity is significantly increasing worldwide. Obesity leads to an increase in many chronic diseases associated with obesity, including Type 2 diabetes, high blood pressure, cardiovascular disease, as well as respiratory disease. Nutrition plays an essential role in preventing obesity, and it has been found that many herbs and spices play a role in improving nutritional status and body weight. Therefore, in this study, we observed the effect of cardamom and cloves on obese rats. Eating cardamom and cloves resulted in a significant reduction in blood lipids and antiobesity properties. Cardamom has shown a greater protective effect on obesity than cloves [11].

The results of our study indicated that there is a significant reduction in BWG from cardamom consumption through the regulation of some obesity-related genes, consistent with previous studies [2, 11–13]. Clove also showed a significant reduction in BWG by inhibiting adipogenesis through blocking the expression. It has been reported for several proteins associated with adipogenesis genes, such as peroxisome proliferator–activated receptor γ (PPARγ), fatty acid synthase (FAS), and Regulatory element binding protein 1 (SREBP-1), which suggests that it has an antiobesity effect. In agreement with previous studies, we also found that cardamom and clove significantly reduced FI and FER, improving overall nutrition [3, 4, 14].

We found a significant reduction in the levels of liver, kidney, heart, lungs, and spleen weight to body weight ratio from eating cardamom and clove by controlling metabolic processes such as FI, adiposity, energy expenditure, thermogenesis of brown adipose tissue (BAT), and browning of white adipose tissue; a HFD and obesity result in the downregulation of sirtuins [11]. It has been suggested that consumption of a certain amount of cardamom caused an increase in sirtuins (from 1.2 to 1.3 ng/mL) in a patient with nonalcoholic fatty liver disease (NAFLD) [15]. Support by clinical trial explained that administration of green cardamom controlled the expression of some diabetes and obesity genes including fat mass and obesity–associated (FTO), carnitine palmitoyltransferase 1A (CPT1A), leptin receptor (LEPR), lamin A/C, and PPAR-γ in women with polycystic ovary syndrome [16].

The study shows the antilipidic properties of cardamom and clove by significantly lowering the levels of TGLs, TC, LDL, and VLDL, while raising HDL. Several studies are consistent with our results regarding the effect of cardamom and clove in regulating body fat mass through a decrease in TGLs, cholesterol, LDL, and VLDL, and an increase in HDL. These effects are attributed to the modification of neural circuits involved in lipolysis in adipose tissue by reducing dyslipidemia, by increasing the rate of cholesterol degradation processes or lipoprotein lipase activity, in addition to an effective reduction in fat absorption from the intestine, and also modifying the oxidative metabolism of mitochondria and axons hypothalamus, pituitary, and adrenal glands. The active compounds found in both cardamom and cloves contribute to their antiobesity properties [2–4, 11–13, 17–22].

Collectively, in agreement with studies, the results of our study provide strong evidence that cardamom and clove extracts improve the nutritional status and exert an antiobesity effect in obese rats, with the contribution of the active compounds present in both cardamom and clove through the regulation of genes related to fat metabolism and modulation of neural circuits in ways that lead to reduced fat accumulation. Moreover, through proposed mechanisms based on literature, cardamom and clove extracts are characterized by active compounds that enhance their antiobesity property. The liver plays a role in increasing mitochondrial activity, regulating fat metabolites, increasing lipolysis and cholesterol degeneration, and decreasing fat absorption.

## 4. Strengths and Limitations

The main strength of the study is that it is considered the first study to compare the effects of cardamom and clove on obesity. In addition, no work has been conducted on a similar dose before and on the possibility of control and commitment to research and the ease of availability of herbs due to the low cost and the flexibility to select between the two herbs based on preference. However, limitations of this study are its relatively short duration and small sample size due to the lack of financial support and the availability of instruments.

## 5. Conclusion

The study showed that cardamom and clove both significantly improve body weight and nutritional status in obese rats, with antiobesity properties through antihyperlipidemic mechanisms (TC, TGLs, LDL, VLDL, and HDL), as well as enhance FI and FER and reduce the relative weights of some organs such as the heart, kidneys, liver, lungs, and spleen. Cardamom extract showed a better effect than clove extract, despite the positive effect of cardamom and cloves on body weight and nutritional status in rats.

## 6. Recommendations

1. Cardamom and clove could be used for weight reduction as natural plants with little or no side effects.
2. Cardamom and clove possess antiobesity properties through antihyperlipidemic mechanisms (TC, TGLs, LDL, VLDL, and HDL) as well as enhance FI and FER and reduce the relative weights of some organs such as the heart, kidneys, liver, lungs, and spleen.
3. Further research is suggested to establish the optimal dose and follow the study in a clinical setting.

## Figures and Tables

**Table 1:** Comparative effect of cardamom and cloves on body weight gain (BWG) in obese rats.

Parameters	BWG (g)
Animal group	Mean ± SE
Control −ve	30.00 ± 0.79^d^
Control +ve	53.00 ± 0.47^a^
Cardamom	41.13 ± 0.35^c^
Cloves	48.18 ± 0.73^b^
LSD	5.31

*Note:* Values denote arithmetic mean ± standard error of the means. Means with different letters (a, b, c, and d) in the same column differ significantly at *p* ≤ 0.05, using a one-way ANOVA test, while those with similar letters are nonsignificant.

**Table 2:** Comparative effect of cardamom and cloves on FI and FER in obese rats.

Parameters	FI (g/day)	FER
Animal group	Mean ± SE	Mean ± SE
Control −ve	11.37 ± 0.08^d^	0.094 ± 0.001^d^
Control +ve	16.13 ± 0.88^a^	0.117 ± 0.002^a^
Cardamom	13.57 ± 0.27^c^	0.108 ± 0.002^c^
Cloves	15.18 ± 0.57^b^	0.113 ± 0.001^b^
LSD	0.05	0.001

*Note:* Values denote arithmetic mean ± standard error of the means. Means with different letters (a, b, c, and d) in the same column differ significantly at *p* ≤ 0.05, using a one-way ANOVA test, while those with similar letters are nonsignificant.

**Table 3:** Comparative effect of cardamom and cloves on the relative liver and kidney weight to body weight ratio in obese rats.

Parameters	Liver (%)	Kidneys (%)
Animal group	Mean ± SE	Mean ± SE
Control −ve	2.57 ± 0.02^d^	0.68 ± 0.01^d^
Control +ve	3.04 ± 0.03^a^	0.87 ± 0.04^a^
Cardamom	2.61 ± 0.01^c^	0.71 ± 0.02^c^
Cloves	2.87 ± 0.03^b^	0.78 ± 0.03^b^
LSD	0.02	0.02

*Note:* Values denote arithmetic mean ± standard error of the means. Means with different letters (a, b, c, and d) in the same column differ significantly at *p* ≤ 0.05, using a one-way ANOVA test, while those with similar letters are nonsignificant.

**Table 4:** Comparative effect of cardamom and cloves on relative heart, lungs, and spleen weight to body weight ratio in obese rats.

Parameters	Heart (%)	Lungs (%)	Spleen (%)
Animal group	Mean ± SE	Mean ± SE	Mean ± SE
Control −ve	0.33 ± 0.02^d^	0.52 ± 0.01^d^	0.23 ± 0.03^d^
Control +ve	0.43 ± 0.05^a^	0.71 ± 0.04^a^	0.61 ± 0.02^a^
Cardamom	0.37 ± 0.04 ^bc^	0.57 ± 0.03^bc^	0.31 ± 0.01^c^
Cloves	0.39 ± 0.03^b^	0.61 ± 0.02^b^	0.41 ± 0.04^b^
LSD	0.02	0.04	0.05

*Note:* Values denote arithmetic mean ± standard error of the means. Means with different letters (a, b, c, and d) in the same column differ significantly at *p* ≤ 0.05, using a one-way ANOVA test, while those with similar letters are nonsignificant.

**Table 5:** Comparative effect of cardamom and cloves on triglyceride and total cholesterol in obese rats.

Parameters	TGL (mmol/L)	TC (mmol/L)
Animal group	Mean ± SE	Mean ± SE
Control −ve	0.76 ± 0.02^d^	1.15 ± 0.02^c^
Control +ve	1.99 ± 0.06^a^	2.08 ± 0.05^a^
Cardamom	0.98 ± 0.01^c^	1.13 ± 0.03^cd^
Cloves	1.17 ± 0.03^b^	1.44 ± 0.04^b^
LSD	0.15	0.13

*Note:* Values denote arithmetic mean ± standard error of the means. Means with different letters (a, b, c, and d) in the same column differ significantly at *p* ≤ 0.05, using a one-way ANOVA test, while those with similar letters are nonsignificant.

**Table 6:** Comparative effect of cardamom and cloves on HDL-c, LDL-c, and VLDL-c in obese rats.

Parameters	HDL-c (mmol/L)	LDL-c (mmol/L)	VLDL-c (mmol/L)
Animal group	Mean ± SE	Mean ± SE	Mean ± SE
Control −ve	0.89 ± 0.02^a^	0.11 ± 0.013^d^	0.152 ± 0.05^d^
Control +ve	0.43 ± 0.06^d^	1.25 ± 0.023^a^	0.398 ± 0.03^a^
Cardamom	0.79 ± 0.01^b^	0.14 ± 0.017^c^	0.196 ± 0.03^c^
Cloves	0.72 ± 0.03^c^	0.19 ± 0.026^b^	0.234 ± 0.07^b^
LSD	0.03	0.02	0.03

*Note:* Values denote arithmetic mean ± standard error of the means. Means with different letters (a, b, c, and d) in the same column differ significantly at *p* ≤ 0.05, using a one-way ANOVA test, while those with similar letters are nonsignificant.

## Data Availability

All data are available in the article.
